# Mucosal Vaccines, Sterilizing Immunity, and the Future of SARS-CoV-2 Virulence

**DOI:** 10.3390/v14020187

**Published:** 2022-01-19

**Authors:** Daniele Focosi, Fabrizio Maggi, Arturo Casadevall

**Affiliations:** 1 North-Western Tuscany Blood Bank, Pisa University Hospital, 56124 Pisa, Italy; 2 Department of Medicine and Surgery, University of Insubria, 21100 Varese, Italy; fabrizio.maggi63@gmail.com; 3 Department of Medicine, Johns Hopkins School of Public Health and School of Medicine, Baltimore, MD 21218, USA; acasade1@jhu.edu

**Keywords:** COVID-19, SARS-CoV-2, neutralizing antibody, BNT162b2, mRNA-1273, IgA, IgG, sterilizing immunity, mucosal vaccines, intranasal vaccine, oral vaccines

## Abstract

Sterilizing immunity after vaccination is desirable to prevent the spread of infection from vaccinees, which can be especially dangerous in hospital settings while managing frail patients. Sterilizing immunity requires neutralizing antibodies at the site of infection, which for respiratory viruses such as SARS-CoV-2 implies the occurrence of neutralizing IgA in mucosal secretions. Systemic vaccination by intramuscular delivery induces no or low-titer neutralizing IgA against vaccine antigens. Mucosal priming or boosting, is needed to provide sterilizing immunity. On the other side of the coin, sterilizing immunity, by zeroing interhuman transmission, could confine SARS-CoV-2 in animal reservoirs, preventing spontaneous attenuation of virulence in humans as presumably happened with the endemic coronaviruses. We review here the pros and cons of each vaccination strategy, the current mucosal SARS-CoV-2 vaccines under development, and their implications for public health.

## 1. Introduction

**Figure 1:**
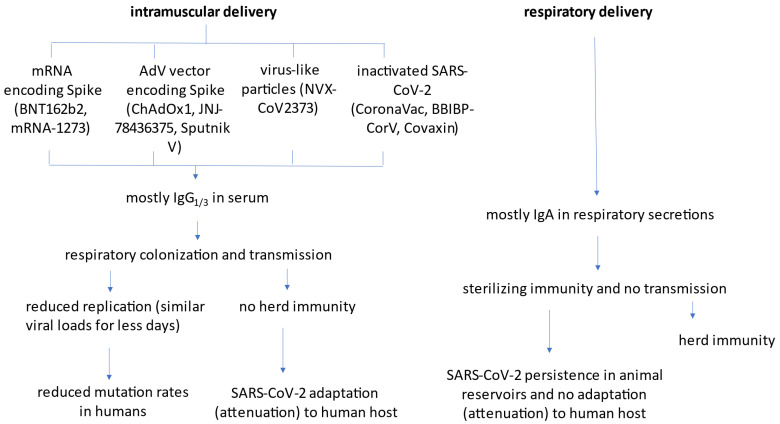
Schematic representation of mechanisms of action of currently approved intramuscular vaccines and next-generation mucosal vaccines.

Currently approved, intramuscularly injected COVID-19 vaccines (summarized in Figure 1) effectively reduce severity of disease and symptomatic cases, but still allow for asymptomatic infection. Most concerning from an epidemiological angle is that these vaccines allow transmission of SARS-CoV-2 and the ability of the virus to replicate in a vaccinated host has the potential for selecting vaccine-resistant variants. The current COVID-19 vaccines primarily induce antibodies of the IgG class (predominantly of IgG_1_ and IgG_3_ subclasses [1]), and little or no respiratory IgA. Although IgG levels are commonly monitored in serum to assess immunity, this isotype, unlike IgA, is not secreted into the mucosal lumen via the polymeric Ig receptor (pIgR), and must rely on passive transport to accumulate at these sites. After systemic administration of IgG, only one out of 1000 molecules in the serum reaches bronchoalveolar lavage (BAL) fluid [2,3,4]. Accordingly, IgG artificially fused to pIgR-binding peptides are more represented in respiratory secretions and more protective in animal-challenge models [5].

Since serum IgG does not effectively penetrate to the mucosal space and serum measurements of vaccine-elicited IgG do not reflect protection from respiratory infection. Nevertheless, after priming with an intramuscular vaccine, subsequent inflammation triggers memory-B-cell migration and secretion of IgA at mucosal sites [6]. Furthermore, any inflammation in the airways enhances serum antibody penetration to the site such that serum immunity can provide early protection in the setting of a developing infection. Hence, intramuscular vaccination does provide some measure of protection in the nasal airways against SARS-CoV-2, as evident by reduction in symptomatic disease after infection.

Asymptomatic infection accounts for one third of SARS-CoV-2-positive nasopharyngeal swabs (NPS), and nearly 75% of cases asymptomatic at the time of the positive NPS will remain asymptomatic [7]. These estimates are likely to be even higher in vaccinees, where breakthrough infections have been demonstrated in asymptomatic subjects [8]. A person might feel fine, but actually harbor replicating SARS-CoV-2 in the nasopharyngeal mucosa and be able to transmit it to others. Given that systematic daily PCR testing would be too invasive and expensive within randomized controlled trials (RCT), several investigators have advocated use of random NPS PCR to improve estimates of vaccine efficacy (VE) against SARS-CoV-2 infection. Such viral-load measures could also be used to estimate efficacy against transmission, assuming the existence of a relationship between viral load and transmissibility [9]. Further supporting this view are data from clinically approved monoclonal antibodies (mAb) to Spike protein—intravenous or subcutaneous injection of anti-SARS-CoV-2 mAbs leads to suboptimal bioavailability in airways, suppressing SARS-CoV-2 replication and lung injury, but allowing robust infection in nasal turbinates in animal models [10]. For this reason, topical delivery of anti-SARS-CoV-2 mAbs is being investigated [11].

We discuss here strategies to prevent asymptomatic carrier status in vaccinees (i.e., how to induce the so-called “sterilizing immunity”), and the theoretical risks of this approach.

## 2. The Difference between Infection and Disease

This simple question is difficult to answer precisely. Vaccines elicit immune responses that are in place when the host encounters the specific microbe. To examine how vaccines work it is important to distinguish between infection and disease. Infection is the acquisition of the microbe by the host while disease is a state of the host–microbe interaction when the host has incurred sufficient damage to affect homeostasis [12]. For most successful vaccines, the measure of efficacy has been reduction in disease since the frequency of infection was not measured. Most current vaccines elicit T-lymphocyte responses and primarily IgG, which provides protection against systemic infection. Robbins et al. proposed that vaccines functioned by neutralizing the infecting inoculum and that this process required a certain amount of antibody [13]. If sites where initial infection might take place are sites accessible to serum IgG then vaccines can prevent infection, but in most cases, vaccines prevent disease by reducing the inoculum, which in turn reduced host damage and the likelihood of disease. In the case of SARS-CoV-2 there is evidence that initial infection and replication occurs in the nasal ciliated cells [14], a site that is not accessible to serum IgG unless there is inflammatory damage to the mucosal tissues that allows transudation of serum proteins to the site. Hence, current vaccines for COVID-19 prevent disease but not infection.

## 3. How Sterilizing Immunity Works

Sterilizing immunity requires timely neutralization of the challenging invader by the humoral immune system. This implies that antibodies are usually more relevant than cell-mediated immunity, at least for viruses and microbes where infection can be directly interfered with by specific antibody. On respiratory mucosae, IgA are the most effective antibody class. Secretory IgA (sIgA), consisting of dimeric IgA, the J chain, and the secretory component, is secreted from glands (e.g., salivary or mammary) and mucosa-associated lymphoid tissue (MALT) onto mucosae, where it neutralizes pathogens. Of interest, sIgA in pre-pandemic human breast milk [15,16] and saliva [17] cross-react with SARS-CoV-2, but whether such heterologous immunity is protective remains unknown. sIgA, with their short half-life of 6.3 days, also represent a useful biomarker for determining recent SARS-CoV-2 infection. Mucosal immunity is also being exploited for passive immunotherapies. For example, several investigators have proposed edible [18] or intranasal [19] egg-derived IgY for passive immunotherapy, and expression of viral antigens in the leaves of edible plants (e.g., lettuce) is also being investigated to induce immunity [20] Similarly, inhalable bispecific single-domain antibodies neutralize Omicron in a mouse model [21].

## 4. Clinically Successful Historical Precedents with Mucosal Vaccines

Many mucosal surfaces are potential sites for vaccine delivery (e.g., conjunctival, nasal, oral, pulmonary, vaginal, and rectal mucosae); however, logistical and cultural reasons have led researchers to focus mostly on oral, nasal, and pulmonary routes [22]. Respiratory mucosal vaccines offer several practical advantages over traditional vaccination approaches, facilitating mass-vaccination campaigns [23] —increased vaccine stability and shelf-life for dry powdered vaccines, painless delivery using disposable inhalers at home [24] (which reduce the requirements for highly trained health-care personnel), and promise eliciting an immune response including sIgA [23].

Examples of clinically successful mucosal vaccines based on attenuated viruses include oral vaccines for gastrointestinal viruses (poliovirus and rotavirus) and intranasal vaccines for respiratory viruses (influenza virus and adenovirus).

The first successful polio vaccine developed by Jonas Salk in 1955 was inactivated and administered intramuscularly. Like the current COVID-19 vaccines, it reduced the risk of illness, but could not prevent infection. In 1960, Albert Sabin developed an oral polio vaccine using three attenuated strains of poliovirus; this approach has led to almost complete eradication of polio worldwide, with as few as 140 cases reported in 2020 in Pakistan and Afghanistan. Nevertheless, recombination of the attenuated strain with wild poliovirus led to local outbreaks caused by type 2 poliovirus included in the trivalent oral vaccine (so-called vaccine-derived poliovirus (cVDPDV2)), with 1089 cases in 26 countries in 2020. This encouraged, after April 2016, the removal of the strain from the trivalent oral vaccine, and finally the return to inactivated vaccines [25,26].

Oral vaccines for rotavirus have been employed for some time in children, demonstrating high efficacy and safety [27,28].

Intranasal influenza vaccines were first used in the 1960s in the former Soviet Union. Licensed intranasal influenza vaccines for humans exploiting nasopharynx-associated lymphoid tissue (NALT) include FluMist/Fluenz™ (MedImmune, Gaithersburg, MD, USA) [29] and the Nasovac™ live attenuated nasal spray manufactured by the Serum Institute of India. The same institute also developed an intranasal, live attenuated influenza virus A(H_1_N_1_), vaccine [30]. None of major flu vaccine manufacturers has engaged with mucosal vaccines yet, so that the majority of flu shots these days are still administered intramuscularly.

Oral vaccines against wild-type adenovirus serotypes 4 and 7 (Ad4 and Ad7) were developed by the National Institutes of Health (NIH) and the US Department of Defense (DoD) in the 1970s. They were originally co-administered [31,32] and then re-formulated in 2011 [33,34,35,36,37]. Both vaccines have increased safety since they do not disseminate systemically, while inducing systemic serum homotypic nAb [34,36,37], and providing >90% efficacy against illness at 12 weeks [33,34,36,37], which is durable for at least 6 years [38].

Hence, vaccines given by mucosal routes are effective and in clinical use. These vaccines provide important precedents for controlling the COVID-19 pandemic and provide great encouragement that similar vaccines can be deployed against SARS-CoV-2.

## 5. IgA Antibodies Play a Key Role in Neutralizing SARS-CoV-2 but Are Rarely Elicited after Intramuscular Vaccination

Natural SARS-CoV-2 infection usually resolves with the appearance of serum monomeric IgA. In fact, much of the neutralizing activity in convalescent plasma resides in the IgM and IgA fraction [39,40]. However, while serum IgG to Spike protein is still present in 92% of the participants after 7 months, serum IgA (and IgM) antibodies decline rapidly after the first month post onset of disease [41,42,43].

Dimeric IgA in secretions similarly have a fundamental role in SARS-CoV-2 neutralization [44]—after infection, very high serum IgA levels develop in patients with severe COVID-19-associated acute respiratory distress syndrome (ARDS) [45], and Spike-specific IgA is dominant in human breast milk [15,16,46].

In general, intramuscularly delivered vaccines induce poor mucosal IgA responses. After the first immunization, BNT162b2 induces anti-Spike IgG_1_, IgG_3_, and IgA_1_ (and sometimes IgG_2_ and IgA_2_) in serum, but only IgG in saliva. One to two weeks after the second dose, IgG levels in saliva and the upper respiratory tract are boosted, while IgA appear in some subjects [47]. Serum IgA has kinetics of induction and time to peak levels (21 days after first dose and 7–10 days after second dose) that are similar to IgG, but a faster decline (>23% reduction from peak at day + 80 after first dose) [48].

For human breast milk the isotype profile differs in the antibody response post-COVID-19 and after vaccination. In contrast to natural infection, immunization with mRNA vaccines during lactation increases anti-receptor-binding domain (RBD) IgA levels in milk [49,50], but not in serum [50]. Unfortunately, only <10% of milk samples from vaccinees have high IgA endpoint titers, but secretory antibody released in milk is both stable and resistant to enzymatic degradation in neonatal mucosal tissues [3,4].

The paucity of IgA after non-mucosal vaccination suggests that systemically vaccinated patients, while mildly symptomatic or asymptomatic, could still become infected with SARS-CoV-2. There is no conclusive evidence yet as to whether asymptomatically infected vaccinees are infectious since theoretically the virions could be immune-complexed and not infectious [51,52,53,54], but the similar viral load seen in NPS from infected vaccinees versus infected naives suggests there is a serious risk of transmission. However, vaccinated individuals who develop symptomatic (e.g., breakthrough) infections are contagious for SARS-CoV-2 [55]. Mild COVID-19 cases in seropositive human vaccinees with positive PCR in different biological matrices (e.g., rectal swab) have also been reported [56], and suggest that the infection in these vaccinees is not limited to the nasopharynx. The case for asymptomatic, vaccinated carriers is of particular concern within hospital settings [8], where patients undergoing clinic and surgical endonasal procedures commonly generate aerosols [57].

Not all systemic vaccines are equally ineffective at inducing mucosal IgA. For example, BNT162b2, but not CoronaVac, induced nasal anti-S1 IgA responses as early as 14 days after the first dose in 72% of subjects, which persisted for up to 50 days after the second dose in 45% of subjects [58], and also induced IgA in breastmilk [59]. Nevertheless, IgA levels in mucosae induced by BNT162b2 or mRNA-1273 are low after the first dose, and decline after the second dose.

## 6. Respiratory Delivery of Vaccines Is Needed to Achieve Sterilizing Immunity against SARS-CoV-2

Animal models involving the related coronaviruses SARS-CoV [60,61,62,63,64,65,66,67,68,69,70] and MERS-CoV [60,71,72,73,74] show that mucosal vaccination induces long-lasting systemic and mucosal immunity. Preclinical evidence from studies in rodents (mice, golden hamsters, and ferrets) have been similarly positive for SARS-CoV-2 (reviewed in [23,75,76,77,78,79] and summarized in Table 1), with the caveat that many studies have not yet been peer-reviewed. At least 14 mucosal vaccines have progressed to the first phase of clinical trials as of 14 December 2021 (Table 2), and several could enter the market in 2022.

Routes other than intramuscular could also lead to antigen dose sparing, which can be relevant to relieving manufacturing bottlenecks during pandemics. While this has been proven for the subcutaneous route [80], it has not been formally proven for mucosal routes.

Although we lack formal studies on the efficacy of oral or inhaled vaccines in human subjects lacking parts of the mucosal immune system (e.g., children after removal of the adenoids and tonsils [81], or after appendectomy), it seems reasonable to assume that the remaining mucosal immune system is enough to induce a response from the fact that there is no historical record that these individuals are more susceptible after receiving other types of vaccines.

## 7. Sterilizing Immunity and the Future of SARS-CoV-2 Virulence

In the case of a vaccine not inducing sterilizing immunity, ongoing transmission is expected to facilitate attenuation to human hosts, although experiences with such “leaky” vaccines in farmed animals have not been conclusive. For example, using Marek disease virus (MDV) in chickens, nonsterilizing vaccines may increase [104] or decrease [105] virulence. Generally, the more a virus circulates, the better should be its adaptation to the host. Evolutionary models suggest that trade-off between virulence and transmissivity maximizes pathogen fitness while reducing virulence, but it is unclear whether this is a universal phenomenon for all viruses [106]. In fact, much depends on the relationship between virulence and transmissibility and the cost of virulence for the microbe in question [107]. For organisms that require virulence for transmission to a new host the capacity for pathogenicity is essential to their survival and attenuation should not necessarily be expected [107]). For SARS-CoV-2 we already know that asymptomatic spread is possible, implying that virulence is not essential for transmissibility. The rapidity of attenuation (decades to millions of years) stems from variables such as lethality and transmission efficiency, making it impossible to draw predictions. While attenuation with circulation among humans is not a universal trajectory, in the past many respiratory viruses have spontaneously attenuated in time, including coronaviruses (e.g., OC-43, which evolved from the causal agent of the 1885–1894 Russian flu pandemic [108] to the virus currently causing common flu). Evidence of mild spontaneous attenuation of human viruses also comes more RNA viruses (e.g., influenza virus A(H1N1) [109,110], dengue virus type 2 [111], and Ebolavirus, from HIV [112,113], although such pathogens are far from being avirulent. The ongoing SARS-CoV-2 pandemic was an unprecedented opportunity to monitor reproductive numbers (*R_t_*) in real-time, and we observed an increase from 1.2 for the original Wuhan strain to 4.0 for the current Delta-plus variant of concern (VOC) [114]: in other words, more virulent and less transmissive variants were rapidly replaced by more transmissive and less virulent variants. Currently, the evolutive process is ongoing with more than 170 Delta sublineages competing each other [115] and another VOC, dubbed Omicron, recently reported [116,117]. Given the increasing spread of attenuated SARS-CoV-2 strains from asymptomatic carriers during lockdown periods, competition of attenuated SARS-CoV-2 strains with the non-attenuated ones has been hypothesized to contribute towards reducing overall virulence [118]. Attenuated viral strains have been a pillar of immunization campaigns for decades, and their intended circulation should not be underestimated.

On the other hand, vaccines causing sterilizing immunity in humans come with the additional risk of preventing spontaneous attenuation by abrogating viral replication and evolution thus pushing the virus into animal reservoirs, where it remains a zoonosis with the possibility of reintroduction into human populations with variants with increased morbidity and lethality (back-adaptation). SARS-CoV-2 is a panzootic virus, and, of interest, all the four current VOCs are able to completely overcome the former Spike restriction for mouse ACE2, and individual VOCs show higher affinities for rat, ferret, and civet ACE2 receptors thanks to N501Y and E484K substitutions in the RBD of Spike.

That said, increased circulation comes with the serious risk of an attenuated lineage reverting to a more virulent one, via single nucleotide mutations, deletions, or recombination. Multiple coronavirus subgenera have a tendency for recombination in low GC genome regions, non-coding regions, edges of genes, and nondisruptive Spike sites [119]. For SARS-CoV-2, two recombinant lineages are already in circulation (XA and XB).

Furthermore, RNA viruses are extremely prone to mutation, exposing them to so-called “lethal mutagenesis” [120], which has been supposed to have caused the sudden disappearance of SARS-CoV-1 [121]. With SARS-CoV-2, this hypothesis has been used recently to explain sudden reduction in incidence of the Delta VOC in Japan [114], but competition from more fit sublineages remains an alternative explanation. Hence, both sterilizing and non-sterilizing vaccines are likely to have a major impact on the course of SARS-CoV-2 virulence evolution for humans, which could pose new future challenges as humanity continues to confront the threat from this virus.

## 8. The Challenge of Vaccine Non-Responders

As with any social need, a compromise is often required between the unmet needs of the vulnerable individuals and the general safety to the immunocompetent population. Poor vaccine responses, persisting after three doses, remain a serious problem for immunocompromised patients [122]. These individuals remain at risk for infection and disease and represent a significant population given the success of modern medicine to treat many oncologic and rheumatologic conditions with therapies that impair immunity. Hence, the problem of non-vaccine-responding immunosuppressed individuals is likely to complicate any efforts to contain or end the pandemic as these hosts remain vulnerable to SARS-CoV-2 and, by replicating the virus for long times, represent an ideal landscape for emergence of viral variants. Prophylaxis with passive immunotherapies is nowadays feasible with s.c. mAbs cocktails (including long-acting antibodies), i.m. hyperimmune sera, or i.v. convalescent plasma. While RCTs are ongoing, there is huge rationale for expecting efficacy from such pre-exposure prophylaxis.

## 9. Are Systemic COVID-19 Vaccines Just “Selfish” Vaccines?

Systemic vaccines unable to provide herd immunity are recognized as “selfish” by generalist press, i.e., they are considered beneficial exclusively for the vaccinee themselves, who is spared from severe disease. Actually, this is a misperception given the obvious social benefits from reduced COVID-19 mortality and morbidity and hospital decongestion, the benefits of systemic vaccines go far beyond the mitigation of COVID-19 course. For example, a shorter duration of viral shedding in NPS (despite peak loads largely similar to unvaccinated cases) leads to reduction of community transmission and to lower probability of within-host mutations. On a wider perspective, the heterogeneity of the SARS-CoV-2 lineages is inversely correlated with rate of vaccination (specifically demonstrated for the Delta VOC on an individual perspective, viral isolates recovered from vaccine breakthrough patients show 2.3-fold lower diversity in known SARS-CoV-2 B cell epitopes in comparison to unvaccinated COVID-19 patients [123]. Hence, the vaccines are already potentially taking a biological toll on viral fitness by reducing its genetic diversity.

## 10. Conclusions

Vaccines remains the best hope for ending the COVID-19 pandemic and reducing mortality. The development of several effective vaccines within the first year of the pandemic was a remarkable accomplishment. Mucosal vaccines were not the primary/first approach taken with SARS-CoV-2 because at the beginning of the pandemic we had poor knowledge of how sterilizing immunity worked against coronaviruses. Nevertheless, vaccines that elicit systemic immunity without mucosal immunity are unlikely to end the pandemic because these prevent disease and not infection, and every case of infection involves viral replication with the opportunity for the emergence of vaccine-resistant variants. Every preventive or therapeutic human intervention against a pathogen creates selective pressure that can lead to the emergence of escape variants [124] and vaccines are no exception. This may apply to competition among lineages as well as accelerated intra-vaccinee evolution. The situation with COVID-19 calls for continued research in vaccine development and given the extent of the global calamity brought by SARS-CoV-2 we anticipate the need for, and development of, a new generation of vaccines that elicit mucosal immunity against multiple viral antigens. Further studies combining post-vaccination monitoring and genetic sequencing of SARS-CoV-2-positive cases are warranted to clarify the effects of altering the natural viral evolution with vaccination campaigns.

## Figures and Tables

**Table 1:** Results of preclinical COVID-19 mucosal vaccines candidates ^1^.

Vaccine		Adjuvant	Schedule	Animal Model	Efficacy	Ref.
Live	Live oral	None	Post-pyloric administration of SARS-CoV-2 by esophagogastroduodenoscopy	Rhesus macaques	Limited virus replication in the gastrointestinal tract and minimal to no induction of mucosal antibody titers in rectal swabs, nasal swabs, or bronchoalveolar lavage.	[82]
Subunit	Recombinant RBD protein	None	Intranasal	Mice	High titers of serum IgG and nAb as well as a significant mucosal immunity	[83]
	Recombinant RBD protein using self-assembling *Helicobacter pylori*–bullfrog ferritin nanoparticles, purified from mammalian cells and assembled into 24-mer nanoparticles	None	Intranasal	Ferrets	No fever, body weight loss, or clinical symptoms; rapid clearance of infectious virus in nasal washes and lungs as well as of viral RNA in respiratory organs.	[84]
	RBD + 2 domains of the viral nucleocapsid protein (N)	Heat-labile enterotoxin B (LTB)	Three-dose vaccination schedule	Mice	Enhanced post-dose-3 nAb, IgG, and IgA production to S- and N-protein-stimulated IFN-γ and IL-2 secretion by T cells	[85]
			Heterologous subcutaneous prime with S1 protein and oral booster	Rats	A single oral booster following two subcutaneous priming doses elicited serum IgG and mucosal IgA levels	
	S1 nanoparticles	IL-15 and TLR agonists	IM-primed/intranasal (IN)-boosted mucosal vaccine	Rhesus macaques	Weaker T-cell and antibody responses, but higher dimeric IgA and IFNa. No detectable subgenomic RNA in upper or lower respiratory tracts	[86]
	S1 protein from the beta variant in PLGA	CP15	Intranasal after WA strain priming 1 year before	Rhesus macaques	Serum- and bronchoalveolar lavage (BAL)-IgG, secretory nasal- and BAL-IgA, and nAb against the original strain and/or beta variant	[87]
Virus-like particles (VLP)	Outer membrane vesicles of *Salmonella typhimurium* conjugated with the mammalian cell culture-derived RBD (RBD-OMVs)	None	Intranasal	Golden Syrian hamster (*Mesocricetus auratus*)	High titers of blood IgG to RBD as well as detectable mucosal responses; no weight loss, lower virus titers in bronchoalveolar lavage fluid, and less severe lung pathology.	[88]
	VLPs displaying RBD (CuMVTT-RBD)	Tetanus-toxin; TLR7/8 ligands.	Intranasal	Mice	Strong RBD- and spike-specific systemic IgG and IgA responses of high avidity; Strong mucosal antibody and plasma cell production in lung tissue	[89]
	Thermostable VLP (e-VLPs) harnessed with variable surface proteins (VSPs) from *Giardia lamblia*, affording them resistance to degradation and expressing pre-fusion stabilized form of S and membrane protein (M) expression	None	I.m. prime-oral boost	Mice and hamsters	Complete protection from a viral challenge; dramatically boosted the IgA mucosal response of intramuscularly injected vaccines.	[90]
Adenoviral vectors	Adenovirus type 5 AdCOVID™	None	Single-dose intranasal	Mice	Elicits systemic and mucosal immunity	[91]
	Human adenovirus type 5	None	Single dose intranasal	mice and ferrets	Complete protection in the upper and lower respiratory tracts.	[92]
	Chimpanzee adenovirus encoding prefusion-stabilized Spike	None	Single dose intranasal	hACE2 transgenic mice	High levels of nAbs, systemic, and mucosal IgA and T cell responses, and almost entirely prevents infection in both the upper and lower respiratory tracts; durable high nAb and Fc effector antibody responses in serum and S-specific IgG and IgA secreting long-lived plasma cells in the bone marrow. At 9 months after vaccination, serum antibodies neutralized SARS-CoV-2 strains with B.1.351, B.1.1.28, and B.1.617.1 spike proteins and conferred almost complete protection in the URT and LRT	[93,94]
	Adenovirus 5- and 19a-vectored vaccines	None	Intranasal vaccinations with adenovirus 5- and 19a-vectored vaccines following a systemic DNA or mRNA priming	Mice	Strong systemic and mucosal immunity; high levels of IgA and tissue-resident memory T cells in the respiratory tract. Mucosal neutralization of VOC was also enhanced. Importantly, priming with mRNA provoked a more comprehensive T cell response consisting of circulating and tissue-resident memory T cells after the boost, while a DNA priming induced mostly mucosal T cells.	[95]
vaccinia vectors	Mucosal homologous plasmid and a heterologous immunization strategy using a plasmid vaccine and a Modified Vaccinia Ankara (MVA) expressing Spike (S) and nucleocapsid (N) antigens.	None	Mucosal	Mice	nAb in serum and bronchoalveolar lavage; induction of Th1 and Th17 responses and polyfunctional T-cells expressing multiple type-1 cytokines (e.g., IFN-γ, TNFα, and IL-2) in the lungs and spleen	[96]
	Pre-fusion-stabilized Washington strain Spike, expressed from a highly attenuated, replication-competent vaccinia virus construct, NYVAC-KC.	None	Intranasal	Mice	Fully protected against disease and death from the mouse-adapted strain of SARS-CoV-2, SARS2-N501YMA30, contains a spike that is also heavily mutated, with mutations at four of the five positions in the Omicron spike associated with neutralizing antibody escape (K417, E484, Q493, and N501).	[97]
Lentiviral vectors	Spike	None	Systemic prime-intranasal boost	hACE2 transgenic mice and golden hamsters	>3 log_10_ decrease in the lung viral loads and reduces local inflammation	[98]
Rhabdoviral vectors	VSV-SARS2(+G) virions generated by G protein trans-complementation	None	Oral	Cynomolgus macaques	Compared to parental VSV-SARS2, G-supplemented viruses were orally active in virus-naive and vaccine-primed cynomolgus macaques, powerfully boosting SARS-CoV-2 nAb titers	[99]
Live attenuated influenza virus vectors	LAIV-CA4-RBD LAIV-HK68-RBD	None	Systemic prime-intranasal boost	K18-hACE2 mice	Higher systemic and mucosal immune responses, including bronchoalveolar lavage IgA/IgG and lung polyfunctional memory CD8 T cells, including against VOC	[100]

^1^ Wording in many of the table cells was taken verbatim from the cited reference to maintain the exact meaning as in the original report.

**Table 2:** Candidate COVID-19 mucosal vaccines in clinical trials and development progress.

Country	Company	Vaccine Name	Technology	Delivery Route	Schedule	Development Progress	NCT Identifier
USA	Altimmune	AdCOVID™	Replication-deficient adenovirus 5 (RD-Ad5)	Intranasal	Single or two-dose intranasal	Phase I (randomized)	NCT04679909
Australia	Tetherex Pharmaceuticals Corporation	SC-Ad6-1	Adenovirus type 6	Intranasal	Single or multiple doses	Phase I	NCT04839042
USA–India	University of Wisconsin–Madison, FluGen and Bharat Biotech	BBV154 (CoroFlu™)	M2-deficient, single replication (M2SR) influenza virus vector	Intranasal	Two doses	Phase I (randomized)	NCT04751682
USA	ImmunityBio, Inc.	hAd5-S-Fusion + N-ETSD	Full-length S and N + enhanced T-cell stimulation domain (ETSD)	Subcutaneous, sublingual, and oral (capsule)	Single dose	Phase I/II (randomized)	NCT04732468 NCT04845191 NCT04591717 [101]
	Vaxart Inc.	VXA-CoV2-1	Non-replicating Ad5 encoding Spike [102]	Oral tablet	One or two doses	Phase II (randomized)	NCT04563702 NCT05067933
China	Institute of Biotechnology, Academy of Military Medical Sciences, PLA of China	Ad5-nCoV	Ad5-nCoV	I.m. prime, intranasal boost	Two doses	Phase I (randomized)	NCT04552366 [103]
Mexico	Laboratorio Avi-Mex, S.A. de C.V.	n.a.	Recombinant Newcastle disease virus (NDV) vectored vaccine	Intranasal prime-i.m. boost	Two doses	Phase I	NCT04871737
UK	Codagenix	COVI-VAC	Live-attenuated virus	Intranasal	Single or two doses	Phase I (randomized)	NCT04619628
UK	University of Oxford	ChAdOx1 nCov-19	Chimpanzee adenovirus expressing Spike RBD	Intranasal	Single dose	Phase I	NCT04816019
USA	CyanVac LLC	CVXGA1-001	Parainfluenza virus 5 (PIV5)-vectored expressing SARS-CoV-2 Spike	Intranasal	Single dose	Phase I	NCT04954287
USA	Meissa Vaccines, Inc	MV-014-212	Live attenuated vaccine against RSV expressing Spike of SARS-CoV-2.	Intranasal	Single or two doses	Phase I	NCT04798001
USA	Symvivo Corporation	bacTRL-Spike	Live *Bifidobacterium longum*, delivering plasmids encoding Spike	Oral	Single dose	Phase I	NCT04334980
New Zealand–USA	Syneos Health–VaxForm LLC	CoV2-OGEN1	n.a.	Oral suspension	Single dose	phase I	NCT04893512
Hong Kong	University of Hong Kong	DelNS1-nCoV-RBD LAIV	Live attenuated influenza virus expressing Spike RBD	Intranasal	Single dose	Phase I	NCT04809389 ChiCTR2000037782

## Data Availability

Data available in a publicly accessible repositories.
